# Exogenous Spermidine Priming Mitigates the Osmotic Damage in Germinating Seeds of *Leymus chinensis* Under Salt-Alkali Stress

**DOI:** 10.3389/fpls.2021.701538

**Published:** 2021-10-13

**Authors:** Chen Hongna, Shi Junmei, Tao Leyuan, Han Xiaori, Lin Guolin, Cheng Xianguo

**Affiliations:** ^1^Laboratory of Plant Nutrition and Biology, Institute of Agricultural Resources and Regional Planning, Chinese Academy of Agricultural Sciences, Beijing, China; ^2^College of Land and Environment, Shenyang Agricultural University, Shenyang, China

**Keywords:** germination, *Leymus chinensis*, polyamine oxidase, salt-alkali stress, spermidine

## Abstract

Spermidine (Spd) is known to protect macromolecules involved in physiological and biochemical processes in plants. However, it is possible that Spd also plays an osmotic regulatory role in promoting the seed germination of *Leymus chinensis* (*L. chinensis*) under salt-alkali stress. To investigate this further, seeds of *L. chinensis* were soaked in Spd solution or distilled water, and a culture experiment was performed by sowing the soaked seeds in saline-alkaline soils. The data showed that the Spd priming resulted in an increase of more than 50% in soluble sugar content and an increase of more than 30% in proline content in the germinating seeds. In addition, the Spd priming resulted in an increase of more than 30% in catalase activity and an increase of more than 25% in peroxidase activity in the germinating seeds and effectively mitigated the oxidative damage to the plasma membrane in the germinating seeds under salt-alkali stress. Moreover, the Spd priming of seeds affected the accumulation of polyamine (PA) and maintained the activities of macromolecules involved in physiological metabolism in germinating seeds exposed to salt-alkali stress. Furthermore, the Spd priming treatment increased the hydrogen peroxide (H_2_O_2_) level to more than 30% and the Ca^2+^ concentration to more than 20% in the germinating seeds, thus breaking the dormancy induction pathways in *L. chinensis* seeds through beneficial hormone enrichment. This study provides an insight into the Spd-mediated regulation pathway during exogenous Spd priming of *L. chinensis* seeds, which mitigates osmotic and oxidative damage and maintains the integrality of the cell lipid membrane. Thus, exogenous Spd priming increases PA oxidase activity and maintains the accumulation of H_2_O_2_. We found that the H_2_O_2_ beneficially affected the balance of Ca^2+^ and hormones, promoting the vigor and germination of *L. chinensis* in response to salt-alkali stress.

## Introduction

Soils with high salinity and alkalinity commonly contain high levels of Na_2_CO_3_ and NaHCO_3_, affecting plant growth and development due to salinization damage (Parihar et al., 2015). Importantly, high salinity-alkalinity in soils severely limits the effective utilization of arable lands with salinization-alkalization. For example, a large tract of highly saline, alkaline land in the Songnen Plain in northeast China is not fully utilized. Moreover, poor parent material characteristics and adverse hydrological conditions aggravate the soil salinization-alkalization in this region, resulting in vegetation degradation and lower plant productivity, especially when overgrazing is not controlled (Horie et al., 2011). *Leymus chinensis* (*L. chinensis*) Trin. Tzvel., one of the most important grassland plants in the Songnen Plain, is a perennial rhizomatous species of the *Poaceae* family containing high levels of proteins, mineral nutrients, and carbohydrates (Lin et al., 2016). Studies have shown that *L. chinensis* has a strong capacity for asexual reproduction and resistance to environmental stresses (Yang et al., 2019). The mature seedlings of *L. chinensis* have a strong tolerance to salt-alkali stress and are the most promising gramineous plant for the rehabilitation of grassland vegetation in soils with salinization-alkalization (Li et al., 2018). However, the low germination rate of *L. chinensis* has been a potential obstacle to its propagation and utility in restoring grassland vegetation in soils with high salinity-alkalinity. Therefore, promoting the germination rates and seedling growth of *L. chinensis* in saline-alkaline soils is important for enhancing the propagation of this grass species to restore the soil ecological balance.

Seed germination and seedling growth are sensitive to abiotic stresses during plant development (Ahanger et al., 2018; Ahmad et al., 2018). Usually, salt-alkali stress interrupts seed germination through osmotic damage and affects endogenous hormones (Ehab, 2016; Shah et al., 2020). Osmotic damage is mainly caused by excess accumulation of Na^+^ because of the presence of higher Na^+^ and Cl^–^ in soils and depletion of K^+^ in plant cells, thus reducing cell osmotic potential (Benito et al., 2014; Ahanger et al., 2019). Oxidative stress is closely associated with the excessive accumulation of reactive oxygen species (ROS) in plants (Miransari and Smith, 2014). Alam et al. (2019) reported that excess ROS causes severe damage to the cell structure or macromolecules, such as proteins, lipids, and nucleic acids, resulting in programmed cell death (PCD) and inhibition of seed germination (Demidchik, 2015).

However, when ROS accumulation is limited to a reasonable level, ROS-mediated signaling regulation can function as an “oxidative window” for seed germination (Bailly, 2019), indicating that ROS homeostasis is a critical factor in seed germination. As a major component of ROS, hydrogen peroxide (H_2_O_2_) has a role in cellular signaling and essential physiological regulation in plants (Mittler, 2017). A previous study has demonstrated that H_2_O_2_ plays a regulatory role in alleviating seed dormancy by balancing hormones in germinating seeds (Wojtyla et al., 2016). In addition to H_2_O_2_, the Ca^2+^ signaling pathway in plants is closely associated with the accumulation of hormones, which usually control stomatal closure in response to drought (Kamala et al., 2016). Unfortunately, most plants cannot maintain the homeostasis of ROS and ions in the germinating seeds under salt-alkali stress, thus delaying seed germination due to oxidative and osmotic damage. Therefore, enhancing the stress tolerance of seeds is important in promoting seed germination and rapid propagation of plants.

To alleviate oxidative and osmotic damage during seed germination, exogenous signal priming agents have been widely used to improve seed germination under abiotic stress (Paparella et al., 2015). For example, polyamine (PA), a promising priming agent, can protect macromolecules and play an important regulatory role in promoting seed germination and plant development under abiotic stresses (Pottosin et al., 2014; Ahanger et al., 2020). Evidence shows that as major components of PA, spermidine (Spd), spermine (Spm), and putrescine (Put) can maintain the metabolic levels of PA by scavenging free radicals, thus improving the osmotic potential, ion homeostasis, and antioxidant enzymatic activities in plant cells under abiotic stresses (Anjum et al., 2017; Alcázar et al., 2020). Both synthesis and oxidation in PA metabolism have demonstrated a protective regulatory role in improving the antioxidant system of H_2_O_2_ induced in *Medicago sativa* (Guo et al., 2014). Previous reports have shown that PA could form a protective mechanism by coordinating with H_2_O_2_, nitric oxide, abscisic acid (ABA), and calcium ion signals in the physiological processes of plants under abiotic stress (Li et al., 2015; Pál et al., 2015). However, there is still little understanding of the regulatory mechanism of Spd-mediated promotion of plant seed germination, especially in *L. chinensis*.

In this study, the seeds of *L. chinensis* were soaked in Spd solution or distilled water and then cultured in soils with high salinity and alkalinity. The physiological response effects of Spd were systematically evaluated by profiling the compatible metabolites, hormones, and osmotic signals to verify whether Spd priming played an osmotic regulatory role in increasing salt-alkali tolerance in germinating seeds of *L. chinensis*. We conducted this study to provide novel insights into the osmotic regulatory pathway of exogenous Spd mediation in germinating seeds and reveal an effective approach for the rapid sexual propagation of *L. chinensis* under abiotic stresses.

## Materials and Methods

### Preparation of Test Materials

*Leymus chinensis* seeds (*L. chinensis* Trin. Tzvel.) were supplied by the Northeast Institute of Geography and Agroecology, China. The soils used for the experiments were collected from the experimental base for saline-alkaline land improvement of Northeast Normal University in Songnen Plain, Jilin Province, China (45°373.21″N122°4958.37″E). The soil samples were mixed to render them homogeneous, placed in a ventilated space for natural drying, and then sieved through a 2-mm width mesh to remove plant fragments and stones. The basic physicochemical characteristics of the soil were mainly represented by a pH of 8.55, soil electrical conductivity of 4.1 ms cm^–1^, total soil nitrogen of 0.8 g kg^–1^, soil total phosphate of 0.23 g kg^–1^, available phosphate of 5.64 mg kg^–1^, and available potassium of 110.28 mg kg^–1^.

### Seed Priming Treatment and Culture

Plump seeds of *L. chinensis* were selected and surface-sterilized with 5% sodium hypochlorite for 5 min, rinsed four times with distilled water, and then soaked in a solution of 1.0 mmol/L^–1^ Spd at 25°C for 12 h. Seeds soaked in distilled water were used as a control. A total of 100 grains of *L. chinensis* seeds with three biological replications were sowed in germination trays containing 80 g of soil with a moisture content of 30%. The germination trays were incubated in a dark germination box at 30°C for 3 days, then transferred to a growth chamber, and further cultured under an alternating cycle of 16 h light and 8 h darkness at an average temperature of 23°C/day and 19°C/night with a relative humidity of 60% and photosynthetic photon flux density of 300 μmol m^–2^ s^–1^ for 14 days.

Seed germination is a process in which the seed radicle breaks through the seed coat (Bewley, 1997). Therefore, we investigated the germination rates and germination energy (GE) on the 17th day (International Seed Testing Association [ISTA], 2010). The germination index (GI) was calculated using the formula of $\text{GI}=\sum \frac{\text{Gt}}{\text{Tt}}$, where Gt represents the number of germinated seeds in *t* days, and Tt represents the number of days corresponding to Gt. Seed vigor index (VI) was determined using the formula of $\text{VI}=\text{GI}\,\text{×}\,\frac{\text{SDW}\,\left(s\,e\,e\,d\,d\,r\,y\,w\,e\,i\,g\,h\,t\right)}{M\,G\,T\,\left(M\,e\,a\,n\,g\,e\,r\,m\,i\,n\,a\,t\,i\,o\,n\,t\,i\,m\,e\right)}$ which was generated using the formula of $\text{MGT}=\sum T\,i\times \frac{N\,i}{\sum N\,i}$ where Ni is the number of new germination seeds in times of Ti (Cao et al., 2019).

### Pharmacological Treatment

The soaked *L. chinensis seeds* with Spd solution were cultured in soils with salinization-alkalization for 3 days and then soaked with distilled water or diphenylene iodonium (DPI) of 1 μmol/L^–1^ or potassium iodide (KI) of 1 mmol/L^–1^ at room temperature for 3 h, respectively. Next, the soaked seeds were used for measuring the Ca^2+^ spatial gradient concentration and fluorescence localization of H_2_O_2_ and Ca^2+^.

### Biomass Measurement

After 17 days of germination, the *L. chinensis* seedlings were collected, and the plant height and fresh weight were measured by standard procedures.

### Measurements of Polyamine and Compatible Metabolites

To understand whether exogenous Spd priming participates in the responses of the germinating seeds of *L. chinensis* to salt-alkali stress, the physiological changes in the germinating seeds of *L. chinensis* were measured after 12 h, 1, 2, and 3 days after sowing the soaked seeds in the soil. After soaking, a portion of the seeds was stored to measure physiological changes, and a total of 15 g seeds were sown in Petri dishes containing saline-alkaline soils and then sampled after 1, 2, and 3 days of culture. All fresh samples were immediately frozen in liquid nitrogen and stored at −80°C for further analyses.

Spermidine, Spm, and Put were extracted and determined using the method described by Liu et al. (2002). Fresh seed samples (0.1 g) from different germination stages were homogenized on ice using a cold solution of 5% perchloric acid (1.5 ml) and centrifuged at 3,000 rpm at 4°C for 10 min. The generated extracts were used to determine the contents of Spd, Spm, Put, and PAs using the double antibody sandwich method of the enzyme-linked immunosorbent assay (ELISA). A 10 μl portion of the recovered supernatant was transferred onto a microporous plate coated with the antibodies of plant Spd, Spm, Put, and PAs that had been incubated in advance at 37°C for 30 min. Next, the microporous plates were cleaned with distilled water five times, 50 μl of horseradish peroxidase (HRP) was added, and the plates were cultured at 37°C for 30 min. Then, the plates were cleaned five times before the addition of 50 μl substrate color developer of 3,3′,5,5′-Tetramethylbenzidine (TMB). A further reaction took place for 10 min and was finally stopped by adding a termination solution. The absorbance values were measured at 450 nm using an enzyme as a marker in a microplate reader (infinite M200 PRO, Tecan, Switzerland), and the contents of Spd, Spm Put, and PAs in the germinating seeds were calculated using the standard curve.

To determine the proline content, fresh samples of germinating seeds (0.1 g) were boiled in 2 ml of 5% sulfosalicylic acid in a water bath at 100°C for 10 min and further heated in the presence of 0.5 ml of glacial acetic acid and acidic ninhydrin (30 min). The absorbance values of the extract were measured at 520 nm using an enzyme as a marker, and the proline content in the seeds was calculated using a standard curve (Bates et al., 1973). Similarly, fresh seed samples (0.1 g) were boiled in 5 ml distilled water in a water bath at 100°C for 30 min twice, then a 0.1 ml extract was supplemented by adding 0.9 ml of distilled water, 0.5 ml of anthrone, and 5 ml of concentrated sulfuric acid for color development and further boiled in a water bath for 30 s. The absorbance values of the extracts were measured at 630 nm using an enzyme as a marker, and the content of soluble sugar in the seeds was calculated using a standard curve (Yemm and Willis, 1954). The malondialdehyde (MDA) content was determined using a modified version of the method of Cao et al. (2010). In detail, 0.2 ml extracts were prepared by extraction with a phosphate buffer (0.05 mol/L^–1^) (pH 7.8) and then transferred into a solution of 0.5 ml trichloroacetic acid (TCA) containing 0.5% (w/v) thiobarbituric acid (TBA), followed by incubation in a water bath at 100°C for 15 min and immediate cooling. After centrifugation at 1,800 × *g* for 10 min, the supernatants were transferred into Corning 96-well plates to measure the optical density (OD) values at 532 and 600 nm using an enzyme as a marker. The MDA content was calculated using an extinction coefficient of 155 mol L^–1^ m^–1^ cm^–1^ and expressed as nmol g^–1^⋅FW.

### Determination of Hormones in the Seeds

An amount of 0.15 g of fresh germinating seeds from each treatment was homogenized in 1 ml of 80% methanol solution and stored at 4°C for 12 h. The sample was treated by ultrasound in an ice bath for 30 min and then centrifuged at 12,000 rpm at 4°C for 5 min. A total of 800 μl of the extract was used to purify the supernatants under reduced pressure at room temperature, then reconstituted by adding 80 μl of 80% methanol solution and loaded in the SPE column (Welchrom C18 SPE, 00559-11001). The sample was then washed twice with 160 μl of distilled water to enrich the plant hormones and then washed twice with 160 μl of 5% methanol aqueous solution. After discarding the washing liquid, the residues were washed four times with 160 μl of methanol solution, and the methanol washing liquid was collected. Further drying was performed under reduced pressure, and 100 μl of methanol was added, then the generated extracts were used to measure the hormone contents by injecting 10 μl of sample into an AB SCIEX-Triple-TOF^®^-5600 + LC/MS/MS system (HPLC, Shimadzu LC30AD System, MS, AB Sciex TripleTOF 5600+).

### Analyses of Enzymatic Activities

The activities of the ADC, ornithine decarboxylase (ODC), spermidine synthase (SPDS), polyamine oxidase (PAO), diamine oxidase (DAO), ascorbate peroxidase (APX), and glutathione reductase (GR) were determined using a Plant ELISA kit (Plant ELISA kit, WeLab, Beijing, China), prepared by the double antibody sandwich of the ELISA. Fresh seed samples (0.1 g) were homogenized in a cold solution of 1.5 ml of 0.05 mol/L^–1^ phosphate buffer (pH 7.8) and centrifuged at 4°C at 3,000 rpm for 10 min or 10,000 rpm at 4°C for 15 min. Then 10 μl of supernatant was transferred into a microporous plate (which had previously been coated with the antibodies of plant ADC, ODC, SPDS, PAO, DAO, APX, and GR) and incubated at 37°C for 30 min, followed by the same procedures as described for the measurements of Spd.

Similarly, fresh seed samples (0.1 g) were homogenized in a 1.5 ml cold solution containing 0.05 mol/L^–1^ phosphate buffer (pH 7.8) and centrifuged at 10,000 rpm at 4°C for 15 min, and the supernatants were used to determine the activities of peroxidase (POD), superoxide dismutase (SOD), and catalase (CAT) at 4°C. SOD (EC1.15.1.1) activity was determined by observing the inhibition of nitroblue tetrazolium (NBT) reduction. In detail, 10 μl crude extracts were mixed with 100 μl reaction mixture containing 5.4 ml of phosphate buffer (0.05 mol/L^–1^, pH 7.8), 0.6 ml of ethylenediaminetetraacetic acid (EDTA)-Na_2_ (30 μmol/L^–1^), 162 ml of 14.5 mol/L^–1^ methionine, 6 ml of 2.25 mol/L^–1^ NBT, and 6 ml of 60 μmol/L^–1^ riboflavin, then transferred into Corning 96-well plates under darkness and then illuminated at 25°C for 15 min. Both non-illuminated and illuminated reactions were performed, and the absorbance values of the reaction solutions were measured at 560 nm using an enzyme as a marker. The SOD activity was calculated using the inhibition of 50% photochemical reduction of NBT as a unit activity. POD (EC 1.11.1.7) activity was determined using the method described previously (Kochba et al., 1977). For POD measurement, the extracts generated by the procedure described above were allowed to react with the mixture containing guaiacol acid, phosphate buffer [0.2 mol/L^–1^ (pH 6.0), and H_2_O_2_ of 30%], and POD activity was measured at 470 nm with a spectrophotometer (ICE3000, Thermo Fisher Scientific, Germany) and expressed as an increase of 0.01 OD value per minute as one enzyme activity unit. CAT (EC1.11.1.6) activity was determined by monitoring the initial rate of H_2_O_2_ disappearance (Dhindsa et al., 1981). Briefly, the extracts generated by the procedure described above were allowed to react with a mixture containing phosphate buffer of 0.15 mol L^–1^ (7.0 pH) and 30% H_2_O_2_. The absorbance values were measured at 240 nm with a spectrophotometer, and the enzyme activity was determined by using a decrease of 0.01 OD value per minute as a unit.

### Measurements of Reactive Oxygen Species, Hydrogen Peroxide, and Electrolyte Leakage

The method used for extracting⋅O_2_^–^ and H_2_O_2_ was the same as that used for extracting antioxidant enzyme crudes. The formation rate of O_2_^–^ was determined by hydroxylamine oxidation (Elstner and Heupel, 1976). A mixture of 0.5 ml phosphate buffer (0.05 mol/L^–1^, pH 7.8) and hydroxylamine hydrochloride (1 ml, 1.0 mmol/L^–1^) was added to 0.5 ml of the enzyme extracts and incubated at 25°C for 1 h. It was then supplemented by adding 1 ml sulfanilic acid (17 mmol/L^–1^ and 1 ml of methyl-naphthylamine 7 mmol/L^–1^) in turn, further incubated at 25°C for 1 h, and centrifuged at 3,000 × *g* at room temperature for 15 min; the resulting supernatants were measured at 530 nm with a spectrophotometer.

The H_2_O_2_ content was determined using an H_2_O_2_ testing kit according to the instructions of manufacturer (Nanjing Jiancheng Bioengineering Institute, Nanjing, China). EL was measured using a conductivity meter (DDS-307A, Shanghai Precision and Scientific Instrument Co., Ltd., Shanghai, China). Fresh seed samples (0.1 g) were immersed in 10 ml of double-distilled water for 24 h to measure the conductivity of the solution (C_1_) and then further boiled in a water bath for 30 min to kill the seeds. The resulting mixture was then used to determine the conductivity of the killed tissues (C_2_). Relative EL was calculated as the percentage of C_1_ over C_2_ (Blum and Ebercon, 1981).

### Determination of K^+^ and Ca^2+^ and Localization of Ca^2+^ in the Sprouting Seeds

The seeds of *L. chinensis* were sampled after 0 or 3 days, and the contents of K^+^ and Ca^2+^ were measured using a modified method (Ali et al., 2012). In brief, fresh seed samples were dried at 80°C until constant weight, and the dried seed samples (0.02 g) were digested in a tube with nitric acid and H_2_O_2_ using a microwave digestion device (MARS6) according to the prescribed procedure. First, the digestion solution was diluted 10-fold, then the contents of K^+^ and Ca^2+^ were measured using an atomic absorption spectrophotometer (ICE3000, Thermo Fisher Scientific, Germany).

### RNA Isolation and Quantitative Real-Time-PCR

Total RNA (0.5 g of seeds with embryos for each sample) was isolated using the total RNA Mini Kit according to the protocol of the manufacturer (RC401-01; Vazyme, China). Both the concentration and purity of the total RNA were determined at a ratio of 260–280 nm. Total RNA was transformed into cDNA by a reverse transcription procedure using HiScript II Q RT SuperMix (R223-01; Vazyme, China), and qPCR was performed according to the instructions of the manufacturer using ChamQ Universal SYBR qPCR Master Mix (Q711-02, Vazyme, China). The qPCR reaction was performed in a mixture of 30 μl containing 2 μl of cDNA and 2 μl of gene-specific primers: the *LcActin* gene (HM623326), Fd: 5′-GCACCCTGTGTTGCTCTACT-3′ and Re: 5′-TACCTTGATCTTCATGCTGCTC-3′ or the *LcSPDS* gene (MT407458, a novel gene in our lab); Fd: 5′-ATGGAGGTTGAGGCGGTGGCG-3′ and Re: 5′-GCCCTTGGCCTCAAATGACCCTCC-3′, and RT qPCR was performed for 40 cycles using an Applied Biosystems (Quant Studio 6 and 7 Flex Real-Time PCR System of Life Technologies) according to a previously described method (Huang et al., 2020). The relative expression levels of the target genes were normalized by using the sample imbibition for 12 h on the 0th day of germination as a reference.

### Fluorescence Localization

Fluorescence localization of H_2_O_2_ in the seed was performed using DCFH2-DA (2, 7-Dichlorodihydrofluorescein diacetate) as described previously (Sakamoto et al., 2005). First, a stock solution of 10 mmol L^–1^ DCFH2-DA was prepared by dissolving the freeze-dried DCFH2-DA powder in a solution of anhydrous dimethylsulfoxide (DMSO). A working solution of 10 μmol L^–1^ was prepared by diluting the stock solution with phosphate-buffered saline (PBS) buffer solution at pH 7.0. After 12 h of soaking, the seeds were cultured for 3 days in soils with salinity-alkalinity, stripped of the bran, and stained in a staining working solution of 10 μmol L^–1^ DCFH2-DA at 30°C for 60 min. The reaction was stopped by transferring the seeds to distilled water. Fluorescence localization of O_2_^–^ in the seeds was measured using a MitoSOX Red fluorescence assay kit (GMS10460.5; GMS, China). Fluorescence imaging of H_2_O_2_ and O_2_^–^ in the seeds was measured using the CHE channel in a Leica Microsystem. Fluorescence localization of Ca^2+^ in the seeds was performed using a cytosolic calcium test kit Fura-2-AM (GMS10152.2; GMS, China). Fluorescence imaging of Ca^2+^ for the seeds was performed using the L5 channel. The integrity of the plasma membrane (PM) in the seed was localized by propidium iodide (PI) as described previously (Sun et al., 2012). All images were taken using a Leica Microsystem (Switzerland, DMC4500). Fluorescent images were processed using the ImageJ software (Xu et al., 2017).

### Seed Viability Detection

Seeds were stained for 12 h to detect seed viability using the 2,3,5-triphenyl-2H-tetrazolium chloride (TTC) method (Ogata et al., 2008). The reaction was stopped by transferring the seeds to distilled water. Seeds were imaged using an Asana mirror (Leica M165FC, Chongqing Leio Instrument Co. Ltd., Germany).

### Non-invasive Micro-Test Technology Assays

Non-invasive micro-test technology (NMT Physiolyzer^®^; Younger, USA LLC, Amherst, MA 01002, United States; Xuyue Science and Technology Co., Ltd., Beijing, China) was used to determine the efflux and influx rates of Ca^2+^, H_2_O_2_, and O_2_ in *L. chinensis* seeds, respectively. The germinating plump *L. chinensis* seeds were picked after 3 days of culture in the saline-alkali soil. Then, the outer bran on the seeds was stripped off and fixed at the bottom of the Petri dish with paper and resin block, and only half the seeds were exposed. The measuring solution was added to the Petri dish, and the seeds were soaked for 20 min. Then, the used measuring solution was discarded, and 5–10 ml of fresh solution was added for sample testing. The test sites of the seeds were identified with a microscope. Specifically, the testing sites for Ca^2+^ and H_2_O_2_ were at the top of the seed embryo and one-half of the seed endosperm, respectively, and the testing site for O_2_ was at the top of the seed embryo. To detect the rates of Ca^2+^, H_2_O_2_, and O_2_, the flux microsensor was placed 10 μm away from the detection site at the seed surface. Each site was tested with four replications for 5–10 min, and the flux rates of Ca^2+^, H_2_O_2_, and O_2_ were directly recorded through the inFluxes V2.0 software (YoungerUSA LLC, Amherst, MA, United States), with a flux unit of mol cm^–2^ s^–1^, where a positive value represents the efflux, and a negative value represents the influx.

### Spatial Imaging of Ca^2+^ Concentration on the Surface of *Leymus chinensis* Seed Embryos

The Ca^2+^ spatial concentration gradient on the surface of living *L. chinensis* seed embryos was detected at a range of 150 μm using Ion Concentration Imaging Technology (NMT Physiolyzer^®^; YoungerUSA LLC, Amherst, MA, United States; Xuyue Science and Technology Co. Ltd., Beijing, China). In detail, the germinating seeds of *L. chinensis* were rinsed with pure water, fixed at the bottom of the Petri dish with filter paper and resin blocks, and then immersed in the test solution containing 0.1 mmol/L^–1^ of CaCl_2_, 0.1 mmol/L^–1^ of KCl, 0.1 mmol/L^–1^ of MgCl_2_, 0.5 mmol/L^–1^ of NaCl, 0.3 mmol/L^–1^ of 2-(N-morpholino) ethanesulfonic acid (MES), and 0.2 mmol/L^–1^ of Na_2_SO_4_ with pH 6.0 for 30 min. Finally, the old test solution was replaced with a fresh testing solution of the same composition for continuous soaking. The background concentration of Ca^2+^ in the measuring solution was detected by an electrode of ø4.5 ± 0.5 μm placed 5 cm from the seed surface. The Ca^2+^ spatial concentration gradient on the surface of *L. chinensis* seeds was determined by an electrode of 4.5 ± 0.5 μm placed at intervals of 5, 35, 65, 95, 125, and 155 μm from the surface of the seed embryo, respectively, and the electrode was maintained at each point for 5 s. Four replications for each sample were automatically detected in four cycles. Distribution images and concentrations of the Ca^2+^ spatial concentration gradient on the surface of *L. chinensis* seed embryos at each spatial detection point were automatically output when the test was finished.

### Transcriptomic Sequencing

For transcriptome sequencing, we used soaked seeds of *L. chinensis* with 1.0 mmol/L^–1^ Spd or water treatment and cultured the soaked seeds for 3 days in saline-alkaline soil under the same culture conditions as described in section “Seed Priming Treatment and Culture.” Then, the germinating seeds in each treatment were separately collected to give three biological replicates and used for total RNA isolation and cDNA library construction. In brief, total RNA of germinating seeds was extracted using a TRIzol Kit (Tiangen Biotech, Beijing), treated with RNase-free DNase I (TaKaRa), and agarose gel electrophoresis was performed to confirm the absence of degradation or contamination. The RNA quality was checked using a NanoDrop spectrophotometer (Implen, Westlake Village, CA, United States), and the RNA integrity was evaluated using an Agilent 2100 Bioanalyzer (Agilent Technologies, Santa Clara, CA, United States). According to the instructions of the manufacturer, cDNA libraries were constructed using a TruSeq Stranded mRNA LT Sample Prep Kit (Illumina). Briefly, mRNA was purified from total RNA using poly T oligo-attached magnetic beads. Fragmentation was conducted using divalent cations under elevated temperature in NEBNext First Strand Synthesis Reaction Buffer (5X). The first-strand cDNA was synthesized using random hexamer primer and M-MuLV Reverse Transcriptase (RNase H), whereas the second-strand cDNA synthesis was synthesized using DNA polymerase I and RNase H.

The cDNA libraries were sequenced on an Illumina Hiseq 4000 platform from the Beijing Allwegene Technology Company Limited (Beijing, China), and paired-end 150 bp reads were generated. Differential expression analysis of two treatments was performed using the DESeq R package (1.10.1), and the genes with an adjusted *P*-value of less than 0.05 were assigned as differentially expressed. Gene ontology (GO) enrichment analysis of the differentially expressed genes (DEGs) was performed using the GOseq R package based on Wallenius non-central hypergeometric distribution (Young et al., 2010). The statistical enrichment of DEGs in the Kyoto Encyclopedia of Genes and Genomes (KEGG) pathways^1^ was performed using the KOBAS method (Mao et al., 2005).

### Statistical Analyses

All data are indicated as means ± SE from three independent biological replications using the ANOVA with the IBM SPSS Statistics software. Significant differences were expressed at the level of <0.05, <0.01, or <0.001 by independent sample *t*-test.

## Results

### Spermidine Priming Improved the Pre-germination Metabolism of *Leymus chinensis* Seeds

A suitable induction agent not only activates the pre-germination metabolic process but also provides protection against seed germination in abiotic stress environments. To explore whether Spd priming plays a regulatory role in improving the germination of *L. chinensis* seeds under salt-alkali stress, a series of responsive changes in physiological and biochemical indicators in *L. chinensis* seeds were measured after 12 h of Spd soaking.

**FIGURE 1:**
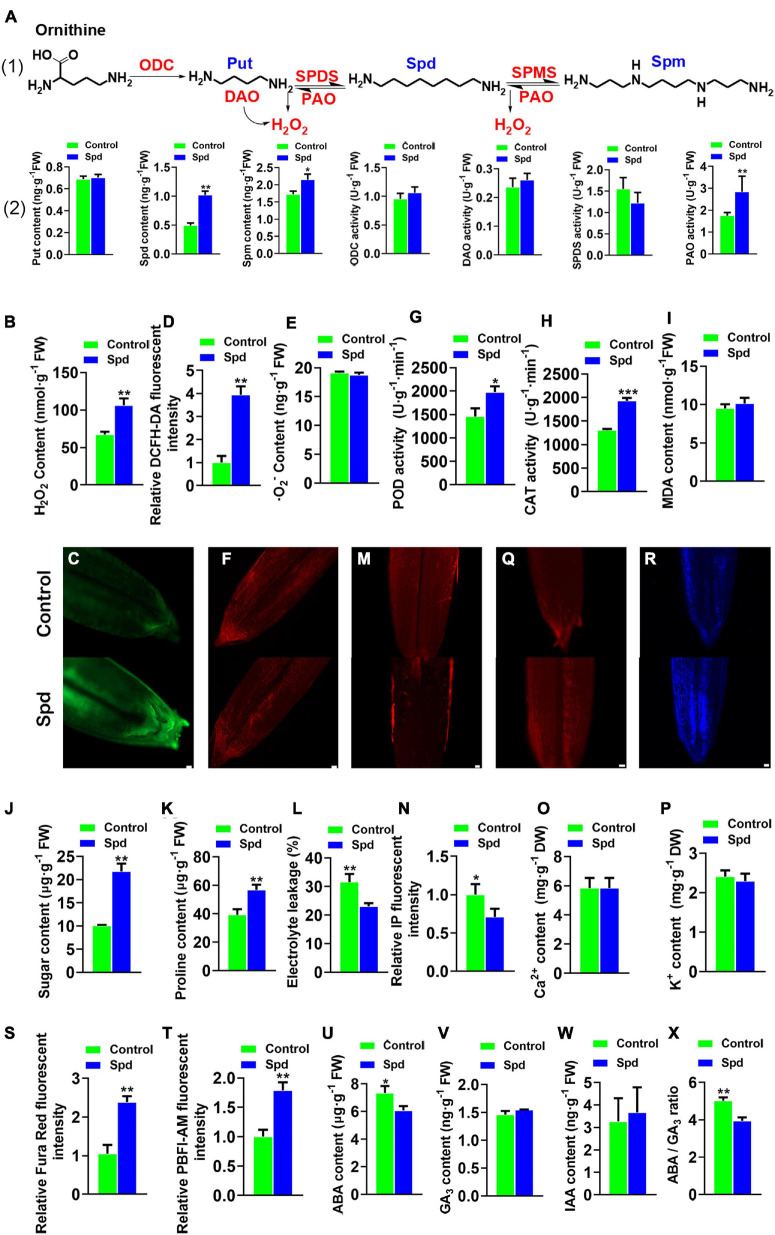
Physiological-biochemical properties of *Leymus chinensis* seeds after 12 h of soaking. **(A)** The polyamine biosynthesis pathway of *Leymus chinensis*, enzymatic activities, relative gene expression, and the contents of Put, Spd, and Spm. **(B)** Content of H_2_O_2_. **(C)** Fluorescence localization of H_2_O_2_, bar = 100 μm. **(D)** Quantification of DCFH2-DA fluorescence intensity. **(E)** Content of O_2_^–^. **(F)** Fluorescence localization of⋅O_2_^–^, bar = 50 μm. **(G)** POD activity. **(H)** CAT activity. **(I)** Content of MDA. **(J)** Content of sugar. **(K)** Content of proline. **(L)** Electrolyte leakages. **(M)** Fluorescence localization of plasma membrane integrity, bar = 100 μm. **(N)** Quantification of PI fluorescence intensity. **(O)** Content of Ca^2+^. **(P)** Content of K^+^. **(Q)** Fluorescence localization of Ca^2+^, bar = 100 μm. **(R)** Fluorescence localization of K^+^, bar = 100 μm. **(S)** Quantification of Ca^2+^ fluorescence intensity. **(T)** Quantification of K^+^ fluorescence intensity. **(U)** Content of ABA. **(V)** Content of GA_3_. **(W)** Content of IAA. **(X)** The ratio of ABA/GA_3_. Vertical bars indicate mean ± SD (*n* = 3), and * or ** or *** indicates a significant difference at the level of *p* < 0.05 or at the level of *p* < 0.01 or at the level of *p* < 0.001, respectively. Put, putrescine; Spd, spermidine; and Spm, spermine; POD, peroxidase; IAA, indole acetic acid; CAT, catalase; ABA, abscisic acid.

Both enzymatic activities and inter-metabolites participating in PA metabolism were measured (Figure 1A). The data revealed that the Spd-primed seeds showed an increase of 51.63% in endogenous Spd, 23.73% in endogenous Spm, and 49.84% in PAO enzymatic activity compared to the control seeds (Figure 1A). At the same time, the H_2_O_2_ content increased by 36.65% (Figures 1B–D). However, the superoxide anion contents representing ROS were not significantly increased (Figures 1E,F), whereas the enzymatic activities of CAT and POD, which scavenge H_2_O_2_, were increased by 32.18 and 26.37%, respectively (Figures 1G,H). Furthermore, the MDA content in the seeds after Spd treatment was not significantly increased compared to that in the control (Figure 1I).

Both soluble sugar and proline are important compatible metabolites that participate in osmotic regulation. Compared with that in the control seeds, an increase of 53.94% in soluble sugar content and 30.72% in proline content was observed in the Spd-primed seeds (Figures 1J,K) and lowered the electrical conductivity by 11.39% (Figure 1L). The Spd-primed seeds clearly demonstrated PM integrity in the germinating seeds (Figures 1L–N). Compared with that in the control seeds, no increase in Ca^2+^ and K^+^ content was observed in the Spd-primed seeds (Figures 1O,P) but a significant enhancement in the fluorescence intensity of Ca^2+^ and K^+^ in the cytoplasm was recorded (Figures 1Q–T), indicating that the Spd priming treatment produced an accumulation of Ca^2+^ and K^+^ in the seed cytoplasm. In addition to the osmotic indicators, the balance between ABA and gibberellins (GA) is a key factor in breaking seed dormancy (Footitt et al., 2011). Therefore, we determined the contents of endogenous hormones ABA, GA, and indole acetic acid (IAA) in the seeds (Figures 1U–X). The results showed that exogenous Spd significantly reduced the accumulation of ABA (Figure 1U) and lowered the ratio of ABA to GA_3_ (Figure 1X).

### Spermidine Priming Promoted the Germination and Viability of *Leymus chinensis* Seeds

**FIGURE 2:**
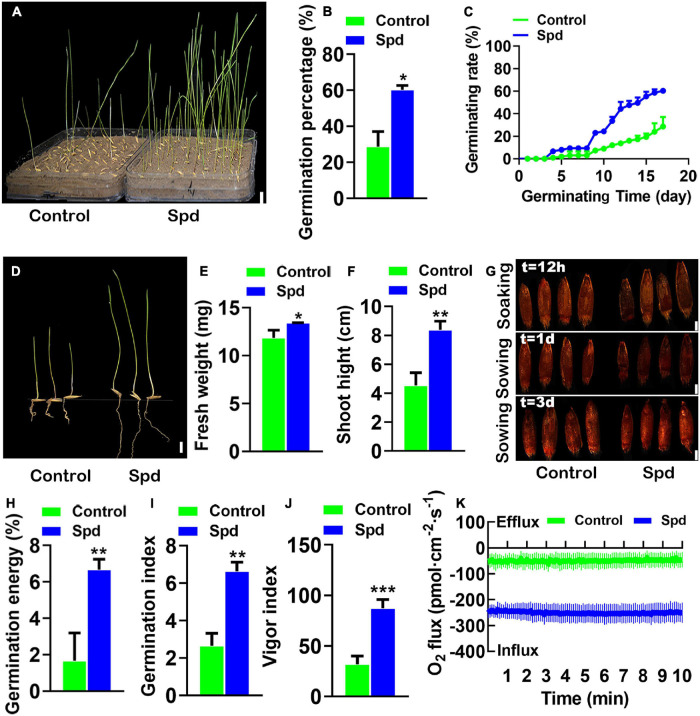
The vigor of the germinating *Leymus chinensis* seeds under salt-alkali stress. **(A)** Germination phenotype of 17 days. **(B)** Germination rates of seeds. **(C)** Time course of seed germination at 25°C. **(D)** The seedling phenotype of 17-day old, bar = 1 cm. **(E)** Fresh weight of 17-day-old seedlings. **(F)** Shoot height of 17-day-old seedlings. **(G)** The seed vigor, bar = 0.1 cm. **(H)** Germination energy. **(I)** Germination index. **(J)** Vigor index. **(K)** Transient net O_2_ flux kinetics. Vertical bars indicate mean ± SD (*n* = 3), and * or ** or *** indicates a significant difference at the level of *p* < 0.05 or at the level of *p* < 0.01 or at the level of *p* < 0.001, respectively.

To confirm the Spd priming effect on seed germination, the soaked seeds of *L. chinensis* with Spd or distilled water were separately sown into the saline-alkaline soils, and the seed germination rates were then investigated (Figure 2A). The germination rates of *L. chinensis* seeds after the Spd priming treatment were increased by 33.66% compared to those in the control seeds treated with distilled water (Figure 2B). The data showed that the final germination speed of the Spd primed seeds was significantly higher than that of the control (Figure 2C). Biomass measurement showed that the Spd priming treatment significantly increased the fresh weights and plant heights of the 17-days-old seedlings (Figures 2D–F). The TTC detection showed that the Spd priming seed treatment not only improved the vigor of the seeds but also had a positive effect on the vigor of the seeds in saline-alkaline soils (Figure 2G). Figures 2H–J shows that the complex parameters representing seed vigor in the Spd priming treatment were notably increased. For example, the GE (Figure 2H), GI (Figure 2I), and vitality index (Figure 2J) in the seeds after the Spd priming treatment were increased by 2. 5–, 2. 7–, and 4-fold, respectively, compared to those in the control seeds. Xin et al. (2013) found that seed vigor was significantly correlated with oxygen influx. Therefore, we used NMT to measure the influx of oxygen in intact seeds. The results showed that the influx of oxygen in the seeds after the Spd priming treatment was significantly higher than that in the control (Figure 2K), and the net oxygen influx in the seeds after the Spd priming treatment was 5-fold higher than that in the control.

### Spermidine Priming Reduced Seed Dormancy Under Salt-Alkali Stress

**FIGURE 3:**
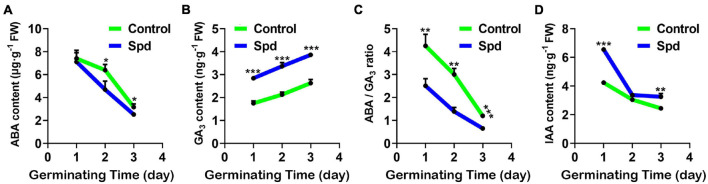
The contents of endogenous hormones. **(A)** Content of ABA. **(B)** Content of GA_3_. **(C)** The ratio of ABA/GA_3_. **(D)** Content of IAA. Vertical bars indicate mean ± SD (*n* = 3), and * or ** or *** indicates a significant difference at the level of *p* < 0.05 or at the level of *p* < 0.01 or at the level of *p* < 0.001, respectively. IAA, indole acetic acid; ABA, abscisic acid.

Salt-alkali stress usually enhances the seed dormancy process (Shu et al., 2017). To investigate whether the strength of seed dormancy is closely related to the accumulation of hormones in the seeds under salt-alkali stress, the accumulation of endogenous hormones, ABA, GA3, and IAA in the germinating seeds of *L. chinensis* was investigated under salt-alkali stress (Figures 3A–D). Compared with the control treatment, the Spd priming treatment significantly increased GA3 content and lowered ABA accumulation in the seeds, thus resulting in a decrease in the ratio of ABA to GA3 under salt-alkali stress (Figure 3C). By contrast, the contents of IAA in the Spd priming treatment significantly increased after 1 or 3 days of sowing under salt-alkali stress (Figure 3D).

### Exogenous Spermidine Indirectly Stimulates Cytoplasmic Ca^2+^ Signals

**FIGURE 4:**
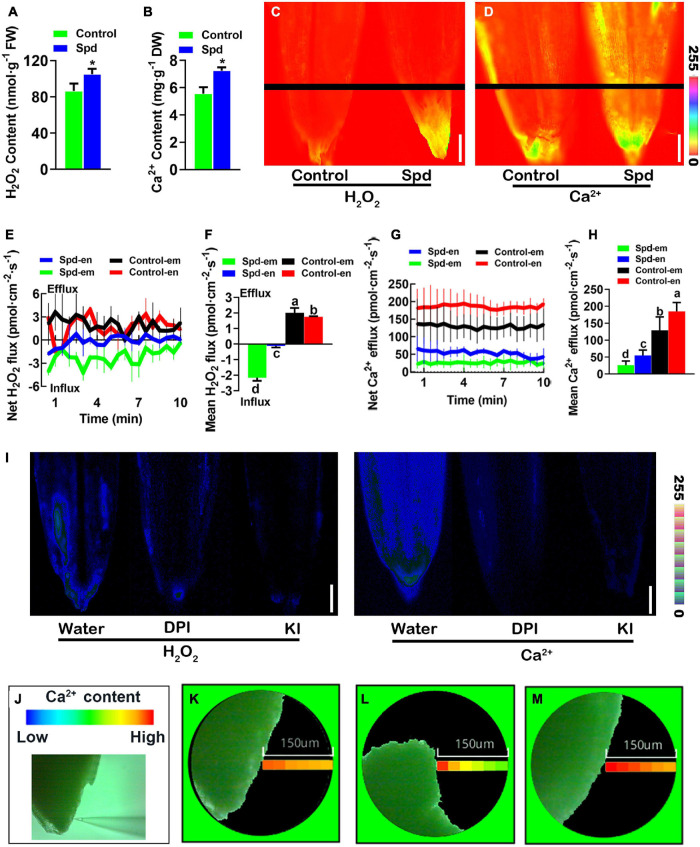
Changes of cytoplasmic Ca^2+^ and H_2_O_2_ signals. **(A)** Content of H_2_O_2_. **(B)** Content of Ca^2+^. **(C)** Representative pseudo-color images, bar = 0.1 cm. **(D)** Representative pseudo-color images of embryo and endosperm, bar = 0.1 cm. **(E)** Transient net H_2_O_2_ flux kinetics. **(F)** Mean rate of H_2_O_2_ flux kinetics. **(G)** Transient net Ca^2+^ flux kinetics. **(H)** Meant rate of Ca^2+^ flux kinetics. **(I)** Representative pseudo-color images of embryo and endosperm, bar = 0.1 cm. **(J)** Detection of the spatial imaging of Ca^2+^ concentration. **(K)** The spatial imaging of Ca^2+^ concentration in the water treatment. **(L)** The spatial imaging of Ca^2+^ concentration in the DPI treatment. **(M)** The spatial imaging of Ca^2+^ concentration in the KI treatment. Vertical bars indicate mean ± SD (*n* = 3), and * indicates a significant difference at the level of *p* < 0.05, and different small letters mean a significant difference at the level of *p* < 0.01.

To investigate whether exogenous Spd regulates the accumulation of H_2_O_2_ and cytoplasmic Ca^2+^ signal levels in the germinating seeds of *L. chinensis* under salt-alkali stress, the contents of H_2_O_2_ and Ca^2+^ in *L. chinensis* seeds were analyzed after 3 days of salt-alkali stress. Compared to the control treatment, the Spd priming treatment resulted in an increase of 30.38% in H_2_O_2_ accumulation and 21.17% in Ca^2+^ accumulation in *L. chinensis* seeds (Figures 4A,B). To further verify this result, we used DCFH2-DA and Fura-2-AM as probes and quantitatively analyzed the relative distribution of H_2_O_2_ and Ca^2+^ in the germinating *L. chinensis* seeds. The results showed that the fluorescence intensity of H_2_O_2_ or Ca^2+^ in the germinating seeds after the Spd priming treatment was significantly stronger than that in the control, and both H_2_O_2_ and Ca^2+^ in the seeds in the Spd priming treatment demonstrated more specific accumulation and high enrichment in embryos (Figures 4C,D). To further characterize the Spd priming effect on the accumulation of H_2_O_2_ and Ca^2+^ in the seed embryo site, we used non-damaging micro-testing technology and measured the flow rates of H_2_O_2_ and Ca^2+^ in the middle part of the seed endosperm and the top of the seed embryo, respectively. Unlike the efflux of H_2_O_2_ in the control treatment, the Spd-primed seeds showed a significant influx of H_2_O_2_ (Figure 4E), and the mean influx rates in the embryos after the priming treatment were significantly higher than those in the seed endosperm (Figure 4F). Both treatments showed an obvious efflux of Ca^2+^ in these tissues (Figure 4G), but the overall efflux rates of Ca^2+^ in the seeds after Spd priming treatment were significantly lower than those in the control, and the efflux rate of Ca^2+^ in the embryos after the Spd priming treatment was significantly lower than that in the seed endosperm (Figure 4H). To verify whether the accumulation of Ca^2+^ in the seeds of Spd treatment is related to the accumulation of H_2_O_2_, we soaked the seeds of *L chinensis* from the Spd priming treatment in water solution, KI, or diphenyleneiodonium chloride (DPI) for 3 h and then performed quantitative fluorescence analyses and spatial imaging of Ca^2+^ concentration. Both KI and DPI treatments resulted in weaker fluorescence intensity of H_2_O_2_ and Ca^2+^ signals exhibited by the seeds than the water treatment (Figure 4I). Spatial imaging of Ca^2+^ concentration in the seed embryos showed that the concentration gradient of Ca^2+^ in the seeds exposed to the microenvironment with water was not obvious (Figure 4J), whereas the concentration of Ca^2+^ near the surface of the seed was slightly higher than that in the environment (Figure 4K). However, the concentration gradient of Ca^2+^ in the seed microenvironment after DPI or KI treatment was significantly greater, and the Ca^2+^ concentration near the seed surface was also significantly higher than that in the environment (Figures 4L,M), indicating that the efflux of Ca^2+^ was notably increased in the DPI and KI treatments.

### Spermidine Priming Maintained the Balance of Hydrogen Peroxide by Affecting Polyamine Oxidase

**FIGURE 5:**
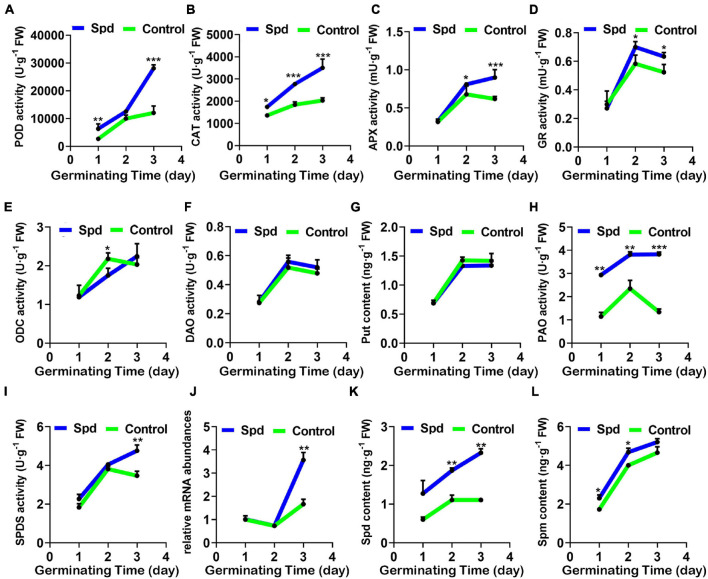
Activity changes of enzymes involved in the regulation of H_2_O_2_ and polyamines of Spd priming mediated. **(A)** POD activity. **(B)** CAT activity. **(C)** APX activity. **(D)** GR activity. **(E)** ODC activity. **(F)** DAO activity. **(G)** Content of Put. **(H)** PAO activity. **(I)** SPDS activity. **(J)** Relative expression of SPDS mRNA. **(K)** Content of Spd. **(L)** Content of Spm. The others are the same as in Figure 1. Put, putrescine; Spd, spermidine; Spm, spermine; CAT, catalase; POD, peroxidase; GR, glutathione reductase; APX, ascorbate peroxidase; ODC, ornithine decarboxylase; DAO, diamine oxidase; PAO, polyamine oxidase; SPDS, spermidine synthase. Vertical bars indicate mean ± SD (*n* = 3), and * or ** or *** indicates a significant difference at the level of *p* < 0.05 or at the level of *p* < 0.01 or at the level of *p* < 0.001, respectively.

To determine whether Spd priming has a regulatory role in maintaining the balance between the production and removal of H_2_O_2_ in *L. chinensis* seeds under salt-alkali stress, the activity changes of the specific enzymes participating in the decomposition of H_2_O_2_ were measured. Compared with the control treatment, the exogenous Spd priming treatment significantly improved the activity of the POD, GR, APX, and CAT enzymes in *L. chinensis* seeds under salt-alkali stress, and the activities of these four enzymes reached a peak after 3 days of sowing. In particular, POD activity in the seeds after the Spd priming treatment demonstrated a 2.99-fold increase compared to that in the control seeds, whereas the enzymatic activities of GR, APX, and CAT in the same treatment only increased by 1. 17–, 1. 24–, and 1.80-fold, respectively (Figures 5A–D).

Since the metabolic pathway of PAs involves the production of H_2_O_2_, we determined the relevant synthetases participating in the PA synthesis (Figures 5E–L). Compared with the control treatment, the Spd priming treatment did not show significant differences in the activities of ODC and DAO or the Put content in the seeds during the germination period (Figures 5E–G). However, the Spd priming treatment showed a 2.49-fold increase in the PA oxidase activity in the seeds, especially after 3 days of sowing compared with the control treatment (Figure 5H). Furthermore, the Spd synthase activity and the relative expression gene encoding Spd synthase showed a 1.19- and 1.94-fold increase, respectively, in the seeds after the Spd priming treatment compared to that in the control germinating seeds after 3 days of culture in the saline-alkaline soils (Figures 5I,J). Similarly, Spd content showed a 2.19-fold increase after 1 day of sowing and a 2.03-fold increase after 3 days of sowing in the seeds after the Spd priming treatment, but Spm content showed only a 1.17-fold increase after 1 day of sowing and 1.15-fold increase after 2 days of sowing compared to that in the control seeds (Figure 5K).

### Spermidine Priming Alleviated the Saline-Alkali Stress Damage to the Plasma Membrane

**FIGURE 6:**
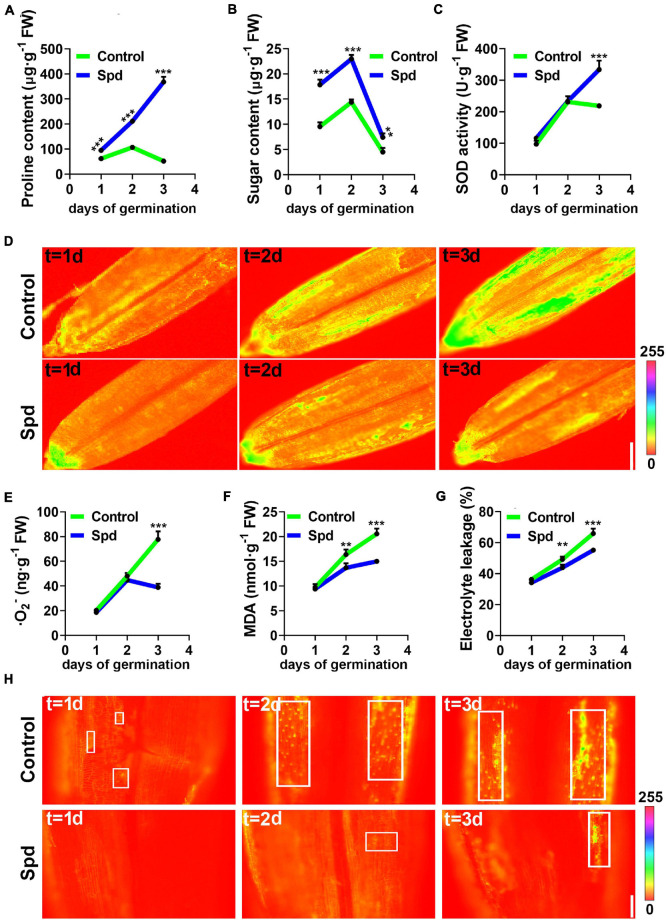
Changes of the osmotic signals under salt-alkali stress. **(A)** Content of Proline. **(B)** Content of sugar. **(C)** SOD activity. **(D)** Representative pseudo-color images of O_2_^–^. bar = 0.1 cm. **(E)** Content of O_2_^–^. **(F)** Content of MDA. **(G)** Electrolyte leakage. **(H)** Representative pseudo-color images, bar = 100 μm. SOD, superoxide dismutase; MDA, malondialdehyde. Vertical bars indicate mean ± SD (*n* = 3), and ** or *** indicates a significant difference at the level of *p* < 0.001.

To better understand whether Spd priming alleviates osmotic damage to the PM, the accumulation of proline, soluble sugar and some indicators reflecting PM damage were analyzed under salt-alkali stress. The data show that the Spd priming treatment led to more proline accumulation in the germinating seeds than in the control under salt-alkali stress. The proline content in the seeds after Spd treatment showed a 1.5-fold increase after 1 day of culture, a 1.9-fold increase after 2 days of culture, and a 7.1-fold increase after 3 days of culture compared to that in the control seeds (Figure 6A). Spd priming significantly increased the accumulation of soluble sugar in the seeds compared to the control treatment, and the soluble sugar content in the seeds after Spd treatment showed a 1.87-fold increase after 1 day of culture, a 1.56-fold increase after 2 days of culture, and a 1.64-fold increase after 3 days of culture as compared to that in the control seeds (Figure 6B). To clarify whether Spd treatment modulates the activity of antioxidant enzymes in germinating seeds, we measured the activity of SOD, which can decompose harmful superoxide anions. The activity of SOD in the seeds after Spd treatment increased by 52.67% after 3 days of culture compared to that in the control seeds (Figure 6C). In addition, quantitative fluorescence results showed that the Spd treatment clearly showed weak fluorescence intensity of superoxide anion in the seeds compared to the control treatment after 3 days of culture under salt-alkali stress (Figure 6D). The superoxide anion content also showed a similar trend to the fluorescence localization (Figure 6E). Compared with the control treatment, the Spd priming treatment resulted in a decrease of 49% in O_2_^–^ accumulation in the seeds of *L. chinensis* after 3 days of culture. Measurements showed that the MDA content in the seeds after Spd treatment decreased by 16.33% after 1 day of culture and by 27.02% after 3 days of culture compared to that in the control seeds (Figure 6F). It is worth noting that the changes in the electronic conductivity of the seeds in the two treatments were utterly consistent with the accumulation of MDA, and the electronic conductivity in the seeds after Spd treatment was significantly lower than that in the control after 1 day of salt-alkali stress (Figure 6G). To investigate whether Spd priming affects the PM of *L*. *chinensis* seeds under salt-alkali stress, we used PI as a soaking agent and fluorescently detected the seeds from the first day of salt-alkali stress. Fluorescence staining showed that the spot number of fluorescent staining was increased in the seeds from the control and revealed a strong fluorescence signal from 1 to 3 days after culture, whereas the spot number of fluorescent staining in the Spd priming treatment clearly demonstrated a weak fluorescence signal (Figure 6H).

### Spermidine Priming Altered the Enrichment Profiles of Differentially Expressed Genes

**FIGURE 7:**
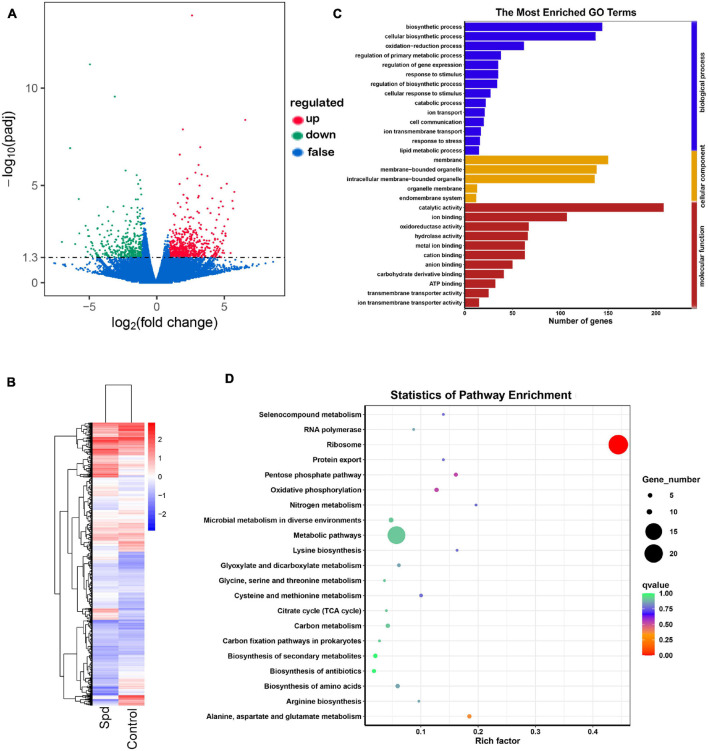
Spd priming altered the enrichment profiles of differentially expressed genes. **(A)** Volcano distributions of DEGs. **(B)** Cluster analysis of differentially expressed genes. **(C)** The GO terms of DEGs. **(D)** Statistics of pathway enrichment of differential genes. DEGs, differentially expressed genes; GO, gene ontology.

To further determine the modulatory role of Spd priming in germinating seeds, transcriptomic analysis was performed using samples after 3 days of culture. Compared with the control seeds, a total of 1,195 genes showed significant differential expression, and a total of 770 genes were upregulated, and a total of 425 genes were downregulated in the germinating seeds after the Spd priming treatment (Figures 7A,B). As shown in Supplementary Table 1, the Spd priming seeds significantly upregulated the DEGs participating in the redox process, which were represented by 4.98-fold upregulation of the gene encoding a quinone oxidoreductase mitochondrial precursor, 4.30-fold upregulation of the gene encoding a 4-hydroxyphenylpyruvate dioxygenase, 3.86-fold upregulation of the gene encoding an alternative oxidase, 3.22-fold upregulation of the gene encoding a heme POD, and 3.06-fold upregulation of the gene encoding an L-xylulose reductase in the germinating seeds under salt-alkali stress compared to the control seeds. Furthermore, the Spd priming seeds evidently resulted in an upregulation of the DEGs participating in cellular respiration in the germinating seeds under culture in the saline-alkaline soils. For example, Spd priming seeds upregulated 3.17-fold of the gene encoding a FAD/NAD(P)-binding domain-containing protein, 2.83-fold of the gene encoding a NAD(P)-linked oxidoreductase, 2.29-fold of the gene encoding an ADP or ATP carrier protein ER-ANT1, and 4.19-fold of the gene encoding a cytochrome c oxidase under salt-alkali stress compared to the control seeds (Supplementary Table 1). In addition, the data show that Spd priming seeds also triggered the upregulation of the DEGs involved in the regulation of osmotic metabolites in germinating seeds under salt-alkali stress. For example, Spd priming seeds upregulated 3.34-fold of the gene encoding a homocysteine methyltransferase, 3.21-fold of alkaline phosphatase, 4.90-fold of the gene encoding an inorganic phosphate transporter similar to a PHO84, 3.13-fold of the pectate lyase, 2.67-fold of the gene encoding a ubiquitin-conjugating enzyme, 1.98-fold of the gene encoding a calmodulin-binding transcription activator (CaMTA), and 1.93-fold of the gene encoding a glycoside hydrolase in the germinating seeds in response to salt-alkali stress, respectively (Supplementary Table 1).

The GO and KEGG metabolic pathway enrichment analyses showed DEGs. GO enrichment analysis showed that the significant GO terms were divided into three subcategories: biological process, cellular component, and molecular function, and the upregulated DEG genes were mainly involved in the biological regulation of metabolic processes and molecular functions. The DEGs participating in biological processes specifically affected the oxidation-reduction process, regulation of primary metabolic processes, catabolic processes, ion transmembrane transport, response to stress, and lipid metabolic processes, whereas the DEGs participating in molecular functions were involved in ion binding, such as calmodulin-binding transcription activator; oxidoreductase activity, such as NAD(P)-linked oxidoreductase; anion binding; carbohydrate derivative binding; and ion transmembrane transporter activity, such as inorganic phosphate transporter (Figure 7C and Supplementary Table 1). The KEGG analysis also showed that the DEGs were mainly involved in oxidative phosphorylation, nitrogen metabolism, such as glutathione S-transferase; citrate cycle (TCA cycle); carbon metabolism, such as 1,3-beta-glucanosyltransferase; and biosynthesis of secondary metabolites and amino acids (Figure 7D and Supplementary Table 1).

## Discussion

As reported previously, PAs with signaling properties play a positive regulatory role in improving the adaptability of plants to abiotic stresses (Pál et al., 2021). Nevertheless, specific components of PAs have different regulatory roles during plant growth. Here, we report that as one of the major components of PA, Spd clearly demonstrates an important regulatory role in improving the salt-alkali tolerance of *L. chinensis* during seed germination. We found that the physiological and osmotic responses of Spd -mediated plants were closely related to ion osmosis, PA synthesis, hormone homeostasis, and H_2_O_2_ signaling in the germinating seeds of *L. chinensis* under salt-alkali stress.

Usually, the damage caused by salinity or alkalinity in germinating seeds is mainly related to osmotic stress. This study shows that exogenous Spd could alleviate osmotic stress by increasing the accumulation of proline and soluble sugar, and this result is consistent with a previous report (Zhang et al., 2016). As compatible metabolites, both proline and soluble sugars play important regulatory roles in maintaining osmotic potentials in plants (Wu et al., 2013), thus alleviating the osmotic damage to the PM of the seeds by protecting the intracellular biochemical reaction. Moreover, the mitigation of osmotic stress to the PM could further prevent oxidative stress in plants (Miguel et al., 2017). Our data show that the Spd priming treatment significantly increased the enzymatic activity of antioxidant SOD and lowered the enrichment of superoxide anion, the content of MDA, and electrical conductivity, indicating that the Spd-mediated osmotic damage mitigation might be associated with light damage to the PM by ROS (Bors et al., 1989; Gill and Tuteja, 2010; Kohli et al., 2019). In this study, the PI staining assay also proved that exogenous Spd plays an important role in protecting the integrity of the cell PM (Crompton et al., 1992). Puyang et al. (2015) reported that exogenous Spd could protect cell membranes not only by reducing the contents of superoxide anions but also by the H_2_O_2_ accumulation profile. Excessive accumulation of H_2_O_2_ usually triggers stress damage to the plant cell, limiting cell growth because of the loss of DNA, proteins, and lipid membranes and causing PCD (Miller et al., 2010). Our results show that although exogenous Spd significantly increased the content of H_2_O_2_, the cell PMs in the germinating seeds of *L. chinensis* were relatively protected under salt-alkali stress, suggesting that exogenous Spd priming of seeds regulates the production and removal of the signal factor H_2_O_2_ in germinating seeds under salt-alkali stress. Transcriptomic sequencing also demonstrated that the upregulated DEGs of Spd-mediated participated in the redox process and cellular respiration in the germinating seeds (Supplementary Table 1), thus affecting the signal distribution, such as H_2_O_2_. As reported previously, H_2_O_2_ is an important signaling molecule that regulates cellular metabolism in plants in response to environmental stimuli (Baxter et al., 2014).

Maintaining a balance between H_2_O_2_ production and removal in cells is particularly important for a positive response to salt-alkali stress. Studies have shown that the CAT-APX-GR enzyme system plays an important regulatory role in removing H_2_O_2_ (Lou et al., 2018; Wu et al., 2018). Our data showed that Spd priming significantly increased the enzymatic activities of the CAT-APX-GR system in the germinating seeds of *L. chinensis*, indicating that Spd priming could activate the enzyme system scavenging H_2_O_2_, thus producing more H_2_O_2_ (Saha et al., 2015). The transcriptomic data also proved that Spd priming altered the oxidation-reduction process and activated the oxidoreductase system. However, increased accumulation of H_2_O_2_ in Spd-mediated cells should be associated with the pathway of H_2_O_2_ production. Fariduddin et al. (2012) reported that exogenous Spd could trigger more H_2_O_2_ production pathways by enhancing the synthesis pathways of PAs, which are catalyzed by PAO and DAO enzymes. Our results show that exogenous Spd priming significantly increased PAO enzyme activity but did not increase DAO enzyme activity, implying that Spd-primed seeds usually generate H_2_O_2_ by activating the reverse synthesis pathway of PAs. Studies have shown that a higher PAO enzymatic activity in the cell produces more POD enzymes by catalyzing the cell wall, thus further catalyzing NADPH and decomposing superoxide anions into H_2_O_2_ (Linkies et al., 2010; Andronis et al., 2014). In our study, POD enzymatic activity in the seeds after Spd priming treatment was significantly higher than that in the control, implying that the increase in the PAO enzymatic activity in the Spd treatment might induce another pathway of H_2_O_2_ accumulation in the germinating seeds of *L. chinensis*.

As a signal sensor, H_2_O_2_ activates the Ca^2+^ channel in guard cells when plants are exposed to environmental stress stimuli (Wu et al., 2020). Our research showed that increased accumulation of Ca^2+^ ions in the germinating seeds under the Spd priming treatment was closely related to the content of H_2_O_2_ under salt-alkali stress because Spd priming simultaneously increased the specific accumulation of H_2_O_2_ and cytoplasmic calcium ions in the seed embryos, and NMT also verified this specific accumulation. Moreover, the Spd priming seed treatment induced the influx of H_2_O_2_, and simultaneously reduced the magnitude of Ca^2+^ efflux in the seeds compared to the control treatment, demonstrating that the influx of H_2_O_2_ plays an important regulatory role in affecting the Ca^2+^ channel in the seed embryos. As previously reported, the flux of Ca^2+^ is closely associated with the accumulation of H_2_O_2_ in plants, and the accumulation of H_2_O_2_ activates the channel of Ca^2+^ influx, thus increasing the accumulation of Ca^2+^ in the cytosol (Niu and Liao, 2016). Furthermore, both the quantitative fluorescence assay and spatial imaging of Ca^2+^ concentration showed a similar result when all the treatments were exposed to water, DPI, or KI, indicating that exogenous Spd priming modulated the cross-membrane transport of ions (Ahmad et al., 2010, 2019). As shown by our transcriptomic analyses, Spd-primed seeds significantly upregulated the expression of the gene encoding a CaMTA DEGs in germinating seeds under salt-alkali stress (Supplementary Table 1).

**FIGURE 8:**
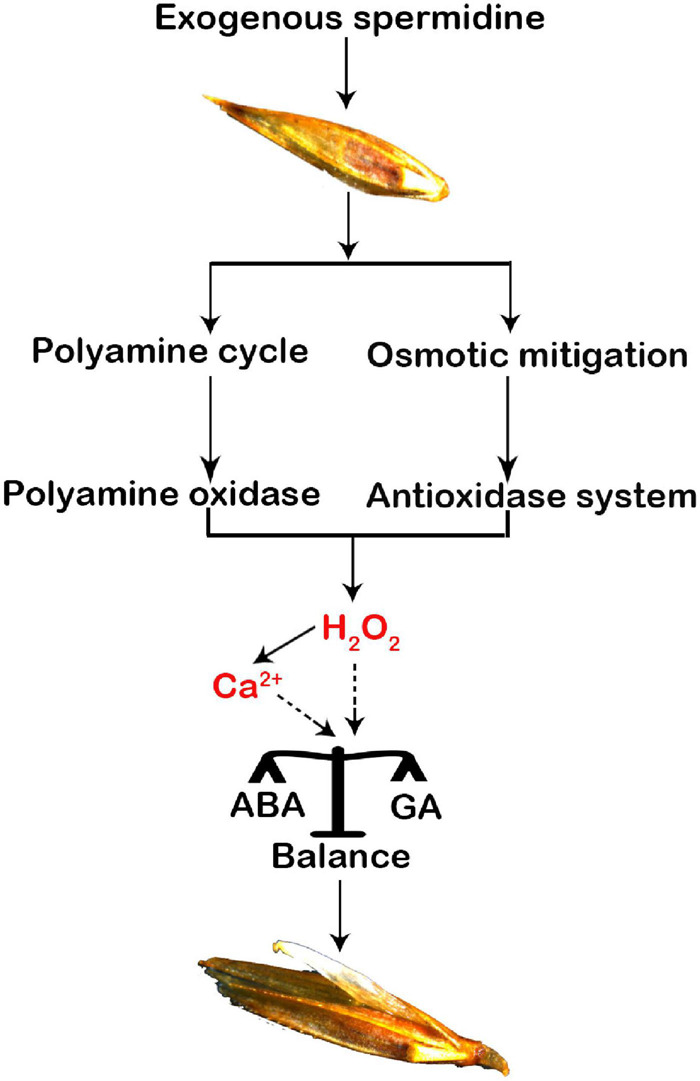
A hypothetical modulation diagram of spermidine-mediated in the seeds of *Leymus chinensis* under salt-alkali stress. A “polyamine circle” was indicated (Pál et al., 2015).

In addition to a previous report that both H_2_O_2_ and Ca^2+^ are important signaling factors that balance the hormones in the seeds, two typical hormones, ABA and GA, have been widely regarded as key signaling factors that promote seed germination (Chen et al., 2020). During seed maturation, the accumulation of endogenous ABA in the seeds induces and maintains seed dormancy (Kim et al., 2013), and high enrichment of GA can promote seed germination (Ju et al., 2020). Our study showed that Spd priming significantly reduced the ABA content induced by salt-alkali stress but significantly increased the content of endogenous GA and IAA, indicating that exogenous Spd priming plays a regulatory role in increasing the accumulation of endogenous hormones in the seeds, thus inhibiting seed dormancy and promoting seed germination (Rajjou et al., 2012). At the same time, exogenous Spd priming also reduced the ratios of ABA to GA, suggesting that exogenous Spd priming could maintain a balance between ABA and GA in the germinating seeds of *L. chinensis*. However, the detailed modulating mechanism is unclear in this study or previous studies. Liu et al. (2010) found that H_2_O_2_ requires the participation of NO signaling to upregulate the genes involved in ABA catabolism (e.g., CYP707A gene), thus decreasing ABA content and affecting the dormancy and germination of *Arabidopsis* seeds. Bahin et al. (2011) suggested that H_2_O_2_ is related to signal transduction and synthesis of GA by activating and breaking dormancy rather than inhibiting ABA signaling. Chen and Guan (2011) reported that both ABA and GA play an essential role in maintaining plant development in response to stress. Under stress conditions, the calcium signal molecule acts to antagonize the effect of ABA in the seeds by inhibiting the expression of the *ABI4* gene, thereby promoting the germination rates of seeds (Kong et al., 2014) because the *ABI4* gene is not only a key transcription factor involved in the ABA response in the seeds but also negatively regulates the gene of GA biosynthesis (Shu et al., 2013). Our results show an obvious increase in the accumulation of H_2_O_2_ and cytosolic calcium ions in the germinating seeds from the Spd priming treatment (Figure 8), suggesting that it is necessary to further clarify whether both H_2_O_2_ and calcium ions might jointly modulate the accumulation profiles of ABA and GA in the germinating seeds in the future study.

## Conclusion

In summary, exogenous Spd priming not only plays an osmotic regulatory role in increasing the accumulation of compatible metabolites but also enhances the PAO enzymatic activity by participating in the PA synthesis pathway in the germinating seeds, thus playing a regulatory role in balancing the production and removal of H_2_O_2_ in the seeds of *L. chinensis* under salt-alkali stress. A beneficial accumulation profile of H_2_O_2_ promoted the accumulation of cytoplasmic calcium ions, and both H_2_O_2_ and cytosolic Ca^2+^ signaling molecules jointly maintained the balance between ABA and GA in the germinating seeds of *L. chinensis*, thereby curtailing the dormancy and improving the vitality and germination rate of the seeds under salt-alkali stress. Beneficial accumulation profiles of endogenous PAs, H_2_O_2_, and Ca^2+^ in the cytoplasm and hormones in the Spd-mediated processes indicate that this study not only reveals novel insights into the regulatory pathway of Spd priming in the germinating seeds of *L. chinensis* under salt-alkali stress but also provides a scientific reference for promoting the seed germination of plants by appropriate utilization of exogenous PA.

## Data Availability Statement

The raw data supporting the conclusion of this article will be made available by the authors, without undue reservation.

## Author Contributions

CH performed the entire experiment and prepared the preliminary manuscript. SJ and TL participated in physiological measurements. LG and HX provided guidance for the experiment. CX designed the entire experiment and corrected the manuscript. All authors contributed to the article and approved the submitted version.

## Conflict of Interest

The authors declare that they have no known competing financial interests or personal relationships that could have appeared to influence the work reported in this paper.

## Publisher’s Note

All claims expressed in this article are solely those of the authors and do not necessarily represent those of their affiliated organizations, or those of the publisher, the editors and the reviewers. Any product that may be evaluated in this article, or claim that may be made by its manufacturer, is not guaranteed or endorsed by the publisher.

## Footnotes

1
http://www.genome.jp/kegg/

## Abbreviations

ABA: abscisic acid
APX: ascorbate peroxidase
ADC: arginine decarboxylase
CAT: catalase
DAO: diamine oxidase
DEGs: differentially expressed genes
FW: Fresh weight
GA: gibberellins
GO: gene ontology
GR: glutathione reductase
IAA: indole acetic acid
KOBAS: KEGG Orthology based annotation system
MES: 2-(N-morpholino) ethanesulfonic acid
NBT: nitroblue tetrazolium
NMT: non-invasive micro-test technology
OD: optical density
ODC: ornithine decarboxylase
PAO: polyamine oxidase
PI: propidium iodide
PAs: polyamines
POD: peroxidase
Put: putrescine
SPDS: spermidine synthase
SOD: superoxide dismutase
Spd: spermidine
Spm: spermine.
