# Supplementary material for: Exogenous Spermidine Priming Mitigates the Osmotic Damage in Germinating Seeds of Leymus chinensis Under Salt-Alkali Stress
Source: Front Plant Sci. 2021 Oct 13;12:701538. doi: 10.3389/fpls.2021.701538 (PMC8548376; doi:10.3389/fpls.2021.701538)
Supplement: Supplementary file 1 [file Table_1.pdf]

Supplementary Table S1 Representatives of significantly up-regulated and down-regulated differentially expressed genes of spermidine-mediated in the transcriptome analysis

Gene-id	log2FoldChange	Description
TRINITY_DN39829_c0_g2	5.0497	mitochondrial thiazole biosynthetic enzyme precursor
TRINITY_DN230257_c0_g1	4.9795	Sulfide:quinone oxidoreductase mitochondrial precurso
TRINITY_DN124884_c0_g1	4.8992	Similar to Inorganic phosphate transporter PHO84
TRINITY_DN55506_c0_g2	4.5718	NADH dehydrogenase subunit
TRINITY_DN29366_c0_g1	4.5094	cysteine dioxygenase
TRINITY_DN31956_c0_g1	4.4877	stearic acid desaturase (SdeA)
TRINITY_DN34665_c0_g1	4.4525	1,3-beta-glucanosyltransferase gel4
TRINITY_DN221604_c0_g1	4.3006	4-hydroxyphenylpyruvate dioxygenase
TRINITY_DN35903_c0_g1	4.2678	GDP-mannose pyrophosphorylase (chromatophore)
TRINITY_DN18328_c0_g1	4.1873	Cytochrome c oxidase
TRINITY_DN229648_c0_g1	4.1841	Aldehyde dehydrogenase
TRINITY_DN241572_c0_g1	3.8652	alternative oxidase
TRINITY_DN3040_c0_g1	3.4761	glutathione S-transferase
TRINITY_DN8238_c0_g1	3.3395	homocysteine methyltransferase
TRINITY_DN67071_c0_g1	3.2223	heme peroxidase
TRINITY_DN173928_c0_g1	3.2136	alkaline phosphatase
TRINITY_DN10212_c0_g1	3.209	the polygalacturonase
TRINITY_DN63_c0_g1	3.1682	FAD/NAD(P)-binding domain-containing protein
TRINITY_DN35587_c0_g1	3.1355	3D-(3,5/4)-trihydroxycyclohexane-1,2-dione hydrolas
TRINITY_DN38775_c0_g1	3.1317	pectate lyase
TRINITY_DN39062_c0_g1	3.0636	L-xylulose reductase
TRINITY_DN41625_c1_g2	2.8287	NAD(P)-linked oxidoreductase
TRINITY_DN12201_c0_g1	2.6663	ubiquitin conjugating enzyme
TRINITY_DN1757_c0_g1	2.4994	epoxide hydrolase
TRINITY_DN65389_c1_g1	2.2928	ADP,ATP carrier protein ER-ANT1
TRINITY_DN59452_c1_g3	1.9799	calmodulin-binding transcription activator
TRINITY_DN45956_c0_g1	1.9297	glycoside hydrolase family 10 protein
TRINITY_DN58507_c2_g1	1.8236	salt-induced protein
TRINITY_DN46309_c0_g2	1.7991	elongation factor 2
TRINITY_DN51949_c2_g1	1.5361	glutathione S-transferase
TRINITY_DN52898_c0_g3	1.4932	salt-induced protein
TRINITY_DN45491_c0_g1	1.274	glycerate dehydrogenase
TRINITY_DN55257_c0_g5	-6.9605	salt-induced protein
TRINITY_DN41190_c0_g1	-5.9772	Glutathione reductase
TRINITY_DN44425_c0_g3	-4.8512	Endo-1,4-beta-xylanase
TRINITY_DN36343_c0_g1	-4.3677	Cys/Met metabolism pyridoxal-phosphate-dependent enzyme
TRINITY_DN46671_c0_g1	-3.5708	Protein arginine N-methyltransferase, putative, partial
TRINITY_DN48984_c5_g6	-2.8974	Peptide transporter PTR2

TRINITY_DN113035_c0_g1	-2.6144	L-ascorbate oxidase-like protein
TRINITY_DN47705_c2_g3	-2.4839	threonine synthase
TRINITY_DN44425_c0_g2	-2.4116	glycosyl hydrolase family 10
TRINITY_DN51714_c1_g3	-2.3304	Cysteine-rich recepto
TRINITY_DN61205_c1_g1	-2.3064	RING-H2 finger protein ATL57
TRINITY_DN46800_c1_g1	-2.1753	putative GPI anchored serine-rich protein
TRINITY_DN53024_c3_g2	-2.0379	leucine rich protein
TRINITY_DN41878_c0_g1	-1.8408	putative oxidoreductase yhhX
TRINITY_DN52376_c0_g1	-1.7411	Lecithin: cholesterol acyltransferase family protein
TRINITY_DN46937_c0_g1	-1.5334	anthocyanin 5-aromatic acyltransferase
TRINITY_DN64941_c0_g2	-1.3333	cellulose synthase
TRINITY_DN59163_c2_g6	-1.2439	alpha-expansin EXPA1
TRINITY_DN51038_c0_g2	-1.2105	acyl-desaturase, chloroplast precursor,putative,expressed
